# Comparative analysis of metagenomic and targeted next-generation sequencing for pathogens diagnosis in bronchoalveolar lavage fluid specimens

**DOI:** 10.3389/fcimb.2024.1451440

**Published:** 2024-08-27

**Authors:** Weijie Sun, Lin Zheng, Le Kang, Chen Chen, Likai Wang, Lingling Lu, Feng Wang

**Affiliations:** ^1^ Clinical Laboratory, The First Affiliated Hospital of Ningbo University, Ningbo, China; ^2^ Infection Technology Platform, Dian Diagnostics Group Co., Ltd., Hangzhou, China

**Keywords:** bronchoalveolar lavage fluid (BALF), pathogen, targeted next-generation sequencing (tNGS), metagenomic next-generation sequencing (mNGS), diagnostics

## Abstract

**Background:**

Although the emerging NGS-based assays, metagenomic next-generation sequencing (mNGS) and targeted next-generation sequencing (tNGS), have been extensively utilized for the identification of pathogens in pulmonary infections, there have been limited studies systematically evaluating differences in the efficacy of mNGS and multiplex PCR-based tNGS in bronchoalveolar lavage fluid (BALF) specimens.

**Methods:**

In this study, 85 suspected infectious BALF specimens were collected. Parallel mNGS and tNGS workflows to each sample were performed; then, we comparatively compared their consistency in detecting pathogens. The differential results for clinically key pathogens were confirmed using PCR.

**Results:**

The microbial detection rates of BALF specimens by the mNGS and tNGS workflows were 95.18% (79/83) and 92.77% (77/83), respectively, with no significant difference. mNGS identified 55 different microorganisms, whereas tNGS detected 49 pathogens. The comparative analysis of mNGS and tNGS revealed that 86.75% (72/83) of the specimens were complete or partial concordance. Particularly, mNGS and tNGS differed significantly in detection rates for some of the human herpesviruses only, including *Human gammaherpesvirus 4* (P<0.001), *Human betaherpesvirus 7* (P<0.001), *Human betaherpesvirus 5* (P<0.05) and *Human betaherpesvirus 6* (P<0.01), in which tNGS always had higher detection rates. Orthogonal testing of clinically critical pathogens showed a total coincidence rate of 50% for mNGS and PCR, as well as for tNGS and PCR.

**Conclusions:**

Overall, the performance of mNGS and multiplex PCR-based tNGS assays was similar for bacteria and fungi, and tNGS may be superior to mNGS for the detection of DNA viruses. No significant differences were seen between the two NGS assays compared to PCR.

## 1 Introduction

Pulmonary infections are prevalent globally, with high morbidity, mortality and healthcare burden (Magill et al., 2014; Shi and Zhu, 2021). Early and accurate pathogen diagnoses are greatly significant, which contribute to make targeted antibiotic therapy and reduction of mortality (Zheng et al., 2021; Diao et al., 2023). Bronchoalveolar lavage (BAL) is considered as a safe, easily performed, minimally invasive and well-tolerated procedure. BALF specimens can facilitate the diagnosis of various lung diseases, including pulmonary infections (Hogea et al., 2020). In clinical settings, the identification of pathogens in BALF specimens is primarily based on traditional microbial culture, microscopic smears and polymerase chain reactions (PCR). Although traditional microbial culture was regarded as the gold standard for pathogens diagnosis of infectious diseases (Fu et al., 2024), it has drawbacks such as lengthy detection cycles, low sensitivity, and challenges in detecting atypical pathogens, viruses, and difficult-to-culture organisms (Lin et al., 2024). Microscopic smears also have low detection rates and few available assay targets (Liu et al., 2022). Also, PCR testing requires specific primers or probes to be pre-designed for microbial pathogens, so this method can only detect known pathogens and has limited ability to detect pathogens in a single assay (Liu et al., 2022; Lin et al., 2024). Due to the aforementioned inherent shortcomings of current microbiological tests, it is difficult to satisfy clinical diagnostic needs.

Recently, next-generation sequencing (NGS) technology, combined with bioinformatics has become a powerful tool for the detection, identification, and analyses of human pathogens, which provides options to overcome the clinical diagnostic challenges of current culture-based and molecular microbiologic techniques (Dulanto Chiang and Dekker, 2020; Maljkovic Berry et al., 2020). Metagenomic next-generation sequencing (mNGS) is a culture-independent, hypothesis-free, unbiased, and comprehensive microbial detection and taxonomic characterization method with high sensitivity, broad pathogens range, and the ability to detect even newly emerging pathogens, which has been demonstrated feasible in the detection and identification of pulmonary infection pathogens (Li et al., 2020; Li et al., 2021; Diao et al., 2023). However, mNGS still faces numerous challenges, such as high costs, great interference of human genes, difficulty in interpreting results, and the inability to conduct DNA and RNA dual-processing detection at the same time (Li et al., 2022; Liu et al., 2022; Lin et al., 2024). To address these challenges, target next-generation sequencing (tNGS) based on targeted amplification technology and high-throughput sequencing technology was developed. At present, tNGS seems to have the advantages of detection sensitivity not affected by the human genome and background microorganisms, lower detection cost, lower sample requirements, easy standardization of workflow, and simultaneous detection of DNA and RNA pathogens (Huang et al., 2021; Lin et al., 2024). However, the performance of tNGS based on multiplex PCR technology for pathogen identification in BALF specimens is not clear yet.

The aim of this study was 2-fold. First, we systematically evaluated the differences in pathogenic diagnostic performance between mNGS and tNGS in BALF specimens, by collecting 85 clinical BALF specimens, and performing mNGS and tNGS detection parallelly, in which tNGS was based on multiplex PCR technology. Second, we evaluated the pathogenic identification value of mNGS and tNGS in BALF specimens by comparing both NGS workflows with PCR.

## 2 Materials and methods

### 2.1 Patients and samples

Clinical specimens used in this study were enrolled from the clinical biobank of The First Affiliated Hospital of Ningbo University in Zhejiang, China, which were collected and retained with the informed consent of the patients. Samples collected between March 2023 and September 2023 that met the inclusion criteria were tested for pathogens by tNGS. Inclusion criteria were as follows: (i) mNGS testing for pathogens identification was complete; (ii) BALF specimens. Exclusion criteria: (i) incomplete clinical records; (ii) samples were contaminated; (iii) residual samples met minimal volume requirements for enrollment. Clinical data of patients were collected through the electronic medical records system. After the mNGS assay, the remaining samples were stored at -80°C until the tNGS assay. At the time of the tNGS assay, enrolled samples have been retained for an average of 30.82 days (range: 0-163 days). This study was approved by the Medical Ethics Committee of The First Affiliated Hospital of Ningbo University (No. 2024-125RS-01) and was conducted according to the principles of the Helsinki Declaration.

### 2.2 mNGS assay

#### 2.2.1 Sample processing and nucleic acid extraction

BALF specimens were collected from patients according to standard procedures. Viscous samples conducted liquefaction treatment. Subsequently, MolYsis™ Basic5 (catalog number D-301-050; Molzym GmbH & Co. KG, Germany), a commercial human DNA depletion kit, was used to remove host DNA. Then, nucleic acid was fully extracted by the Magnetic Pathogen DNA/RNA Kit (catalog number NG550; Tiangen Biotech (Beijing) Co., Ltd, China) following the manufacturer’s instructions. The concentration of extracted DNA was measured using a Qubit™ double-stranded DNA (dsDNA) high-sensitivity (HS) assay kit (catalog number Q32854; Thermo Fisher Scientific Inc, USA).

#### 2.2.2 Library preparation and sequencing

DNA libraries were constructed using the VAHTS Universal Plus DNA Library Prep Kit for MGI (catalog number NDM617; Vazyme, China) with the 2 ng initial input. The quality control (QC) of DNA libraries was carried out using an Agilent 2100 bioanalyzer (Agilent Technologies Inc., USA) to assess DNA concentrations and fragment size. DNA libraries with a main peak of 240 bp-350 bp and concentration greater than 1ng/μL passed the QC process. Qualified libraries were pooled together for denaturation and circularization to generate a single-stranded DNA circle (ssDNA circle). Then, DNA nanoballs (DNB) were generated via rolling circle replication (RCA). Finally, prepared DNBs were loaded onto the sequence chip and sequenced on a BGISEQ platform for single-end 50-bp sequencing to generate 10 ∼to 20 million reads for each library.

#### 2.2.3 Bioinformatic analysis

Pathogen detection by mNGS was performed using an in-house bioinformatics pipeline. After sequencing, Fastp v0.23.4 was used to remove low-quality reads, adapters, and short reads to obtain clean data for further analysis (Chen et al., 2018). BWA (Burrows-Wheeler Aligner) v0.7.17-r1188 was used to identify human sequences by mapping clean data to three human reference genomes, including hg38, T2T-CHM13, YH1 (Li and Durbin, 2009). And human sequences were excluded by Samtools v1.6. Our in-house genome database consisted of 8188 microbes genomes with 4,973 bacterial species, 431 DNA viral species, 678 RNA viral species, 1843 fungal species, and 263 parasites (**Supplementary Table 1**). To construct the microbial genome database, firstly, we constructed a pathogens list according to the following three references: (i) official information including prioritizing diseases for research and development in emergency contexts by WHO (https://www.who.int/activities/prioritizing-diseases-for-research-and-development-in-emergency-contexts), and National CDC Catalogue of Human Pathogenic Microorganisms published by the official Chinese; (ii) books including Johns Hopkins ABX Guide (https://www.hopkinsguides.com/hopkins/index/Johns_Hopkins_ABX_Guide/Pathogens), Harrison’s Infectious Diseases (3^rd^ Edition), and Manual of Clinical Microbiology (12^ed^ Edition) (https://www.clinmicronow.org/doi/book/10.1128/9781683670438.MCM); (iii) clinical case reports or research articles published in current peer-reviewed journals. Secondly, the microbial genome databases were downloaded from RefSeq and WGS in the National Center for Biotechnology Information (https://www.ncbi.nlm.nih.gov/). After human reads filtering, the remaining sequences were aligned to our microorganism genome database by BWA. Then, by processing the mapped data with in-house scripts, all microorganisms contained in the sample were identified. The results were further verified by BLAST.

#### 2.2.4 Threshold criteria and result interpretation

Raw sequencing read counts for individual microorganisms were normalized to produce the reads per twenty million (RPTM), so that samples with different sequencing depths or reads are comparable. To identify background microorganisms, negative controls (NTC) were established for each batch of experiments. Furthermore, we constructed a clinical grades-based filtering system for interpretation to exclude the effects of redundant organisms. Specifically, based on the above references, we classified pathogens in our internal genome database into four grades, with clinical importance IV>III>II>I (**Supplementary Table 1**). Different interpretation rules were developed for pathogens of different clinical importance grades (I~IV) and biological taxonomy (bacteria, viruses_DNA, viruses_RNA, fungi, and parasites), which were mainly realized by different thresholds of microorganisms-specific RPTM, species-rank within the genus, genus relative abundance, genus-rank, RPTM-r (RPTM-r was defined as the RPTM_sample_/RPTM_NTC_). The process did not report RNA viruses. Suspected background microorganisms were excluded from provisional mNGS test reports. Pathogens found in BALF specimens were identified by two experienced laboratory clinicians based on clinical features, smears of specimens, and other microbiology testing.

### 2.3 tNGS assay

The multiplex PCR-based targeted next-generation sequencing technology was performed to identify targeted pathogens. This testing included 231 clinically important or relevant pathogens, based on the aforementioned official information, book, clinical case reports, or research articles (**Supplementary Table 2**). By taxonomy, the panel included 108 bacteria, 17 DNA viruses, 54 RNA viruses, 46 fungi, and 6 parasites. The production and testing of the panel were performed in-house by Beijing Genskey Technology Co., Ltd.

BALF specimen processing and nucleic acid extraction methods were consistent with that of mNGS. This tNGS detection method, was divided into two tubes of DNA and RNA, respectively, for multiple amplification. For RNA viruses, no more than 1μg of nucleic acid product was taken for reverse transcription and cDNA synthesis sequentially. Multiplex PCR amplification was performed separately for gDNA and cDNA to enrich the target gene sequences. Then, the products of the two tubes were combined to purify. By adding sequencing adapters and barcode sequences for sample identification, pathogen sequencing libraries were obtained. Libraries QC, pooling, DNB preparation, sequencing platform, and parameter settings are the same as mNGS assay, the only difference is that a single sample generates 0.5 million reads. After sequencing, the raw reads were filtered, short reads or non-specific primer binding was removed, and then, clean reads for identification were obtained. Subsequently, pathogen species in the samples were identified by sequence read counts from sequence alignment. Similarly, we established a set of interpretation rules for the tNGS assay, which were mainly realized by indicators such as microorganisms-specific RPM (RPM was defined as the reads per million), RPM-r (RPM-r was defined as the RPM_sample_/RPM_NTC_), and primer amplification status. The interpretation of the tNGS results was also done by two experienced laboratory clinicians based on clinical features, smears of specimens, and other microbiology tests.

### 2.4 Orthogonal confirmation of mNGS and tNGS results

When the results of mNGS and tNGS assays were inconsistent, samples underwent orthogonal testing utilizing specific PCR. In this study, PCR was used for the detection of clinically critical pathogenic microorganisms, including: (i) *Mycobacterium tuberculosis complex*; (ii) non-tuberculous *Mycobacteria* such as *M. avium*, *M. chelonae*, *M. intracellulare*, etc; (iii) fungi including *Aspergillus flavus*, *A. fumigatus*, *A. niger*, *Cryptococcus neoformans* and *Pneumocystis jirovecii*. Orthogonal experiments could only be carried out if the residual sample met the minimum volume requirements of the PCR assay.

### 2.5 Statistical analysis

IBM SPSS v26.0 (SPSS Inc., USA) was used for the data analysis. Categorical variables were described as frequencies and percentages. In contrast, continuous variables conforming to a normal distribution were expressed as mean ± standard deviation. Those with a nonnormal distribution were presented as the median and interquartile range (IQR). Comparative analyses were conducted by McNemar’s chi-squared test and Fisher’s exact test. A P-value of less than 0.05 was regarded as statistically significant.

## 3 Results

### 3.1 Demographic characteristics

A total of 85 BALF specimens from 83 patients were initially enrolled in this study according to the inclusion and exclusion criteria. Subsequently, 2 samples failed the QC process of the tNGS workflow. Finally, 83 samples were included in further analyses. For the final enrolled patients, except for one patient who had three samples (Patient 20, Sample #20, 35, 51), the remaining 80 patients had only one sample. The ages of this cohort ranged from 15 to 87 years old, with a mean age of 58.7 years, in which 1 case (1.2%) was 20 years of age or younger and 42 cases (51.9%) were older than 60 years of age. Approximately 58.0%% (47/81) of the cases were male (**Table 1**; **Supplementary Table 3**).

**Table 1:** Patient Demographics.

Characteristic	
Age	
Mean yr	58.7
Distribution no. (%)	
1-20 yr	1 (1.2)
21-60 yr	38 (46.9)
≥60 yr	42 (51.9)
Gender no. (%)	
Female	34 (42.0)
Male	47 (58.0)

### 3.2 Pathogen identification by mNGS and tNGS

The microbial detection rates of mNGS and tNGS were 95.18% (79/83) and 92.77% (77/83), respectively, and the difference was not statistically significant (P=0.625).

**Figure 1:**
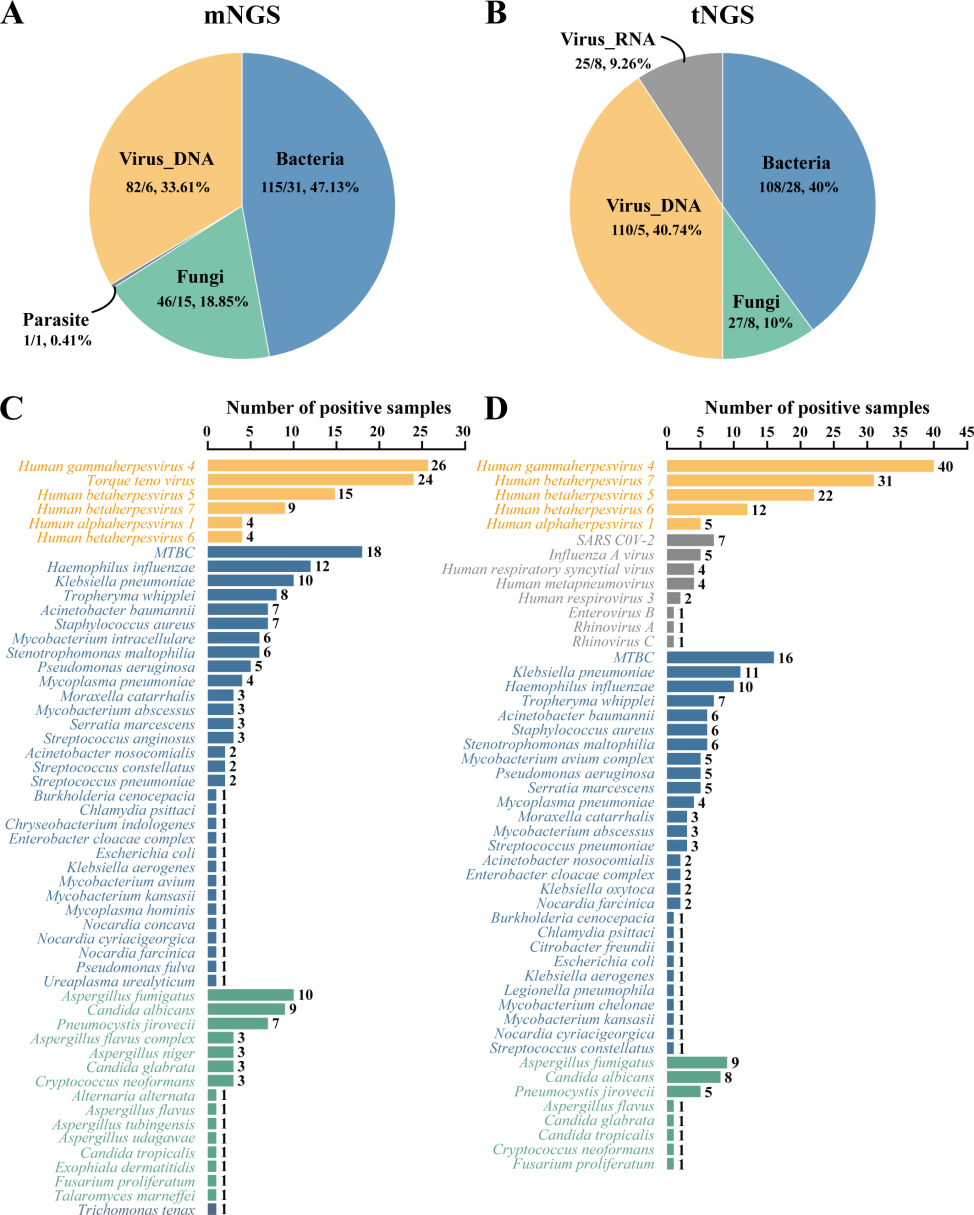
Pathogen identification by mNGS and tNGS workflows. **(A, B)** show the distribution of pathogens detected by mNGS and tNGS assays by categories, respectively. Before the slash is the number of strains detected, and after the slash is the type number of species detected. **(C, D)** show the pathogen detection results of mNGS and tNGS assays, respectively. *MTBC*, *Mycobacterium tuberculosis complex*.

The pathogen detection results of mNGS and tNGS workflows are shown in **Figure 1**, with bacteria and DNA viruses mainly detected. A total of 244 pathogens were detected by the mNGS workflow. Bacteria were from 31 species, accounting for 47.13% (115/244) of detections, with *Mycobacterium tuberculosis complex* (*MTBC*, 18/115, 15.65%), *Haemophilus influenzae* (12/115, 10.43%), *Klebsiella pneumoniae* (10/115, 8.70%) being the most highly detected. DNA viruses belonged to 6 species, accounting for 33.61% (82/244) of detections, while detection rates of *Human gammaherpesvirus 4* (26/82, 31.71%), *Torque teno virus* (24/82, 29.27%) and *Human betaherpesvirus 5* (15/82, 18.29%) were the highest. Fifteen fungi species with a total of 46 strains were detected, accounting for 18.85% (46/244). *Aspergillus fumigatus* (10/46, 21.74%), *Candida albicans* (9/46, 19.57%) and *Pneumocystis jirovecii* (7/46, 15.22%) had the highest detection rate. Parasites were detected in 1 case of *Trichomonas tenax*, accounting for 0.41% (1/244) (**Figures 1A, C**).

In contrast, the tNGS workflow identified 270 potential pathogens from 83 BALF specimens. Bacteria were from 28 species, accounting for 40.00% (108/270), while *MTBC* (16/108, 14.81%), *K. pneumoniae* (11/108, 10.19%) and *H. influenzae* (10/108, 9.26%) had the highest detection rate. DNA viruses were belonging to 8 species, accounting for 40.74% (110/270). The detection rates of *Human gammaherpesvirus 4* (40/110, 36.36%), *Human betaherpesvirus 7* (31/110, 28.18%) and *Human betaherpesvirus 5* (22/110, 20.00%) were the highest. The RNA viruses were from 8 species, accounting for 9.26% (25/270). The detection rates of *Severe acute respiratory syndrome coronavirus 2* (7/25, 28.00%) and *Influenza A virus* (5/25, 20.00%) were the highest. Eight fungi species with a total of 27 strains were detected, accounting for 10.00% (27/270), with *A. fumigatus* (9/27, 33.33%), *C. albicans* (8/27, 29.63%) and *P. jirovecii* (5/27, 18.52%) being the most highly detected (**Figures 1B, D**).

### 3.3 Comparison of mNGS and tNGS results

**Figure 2:**
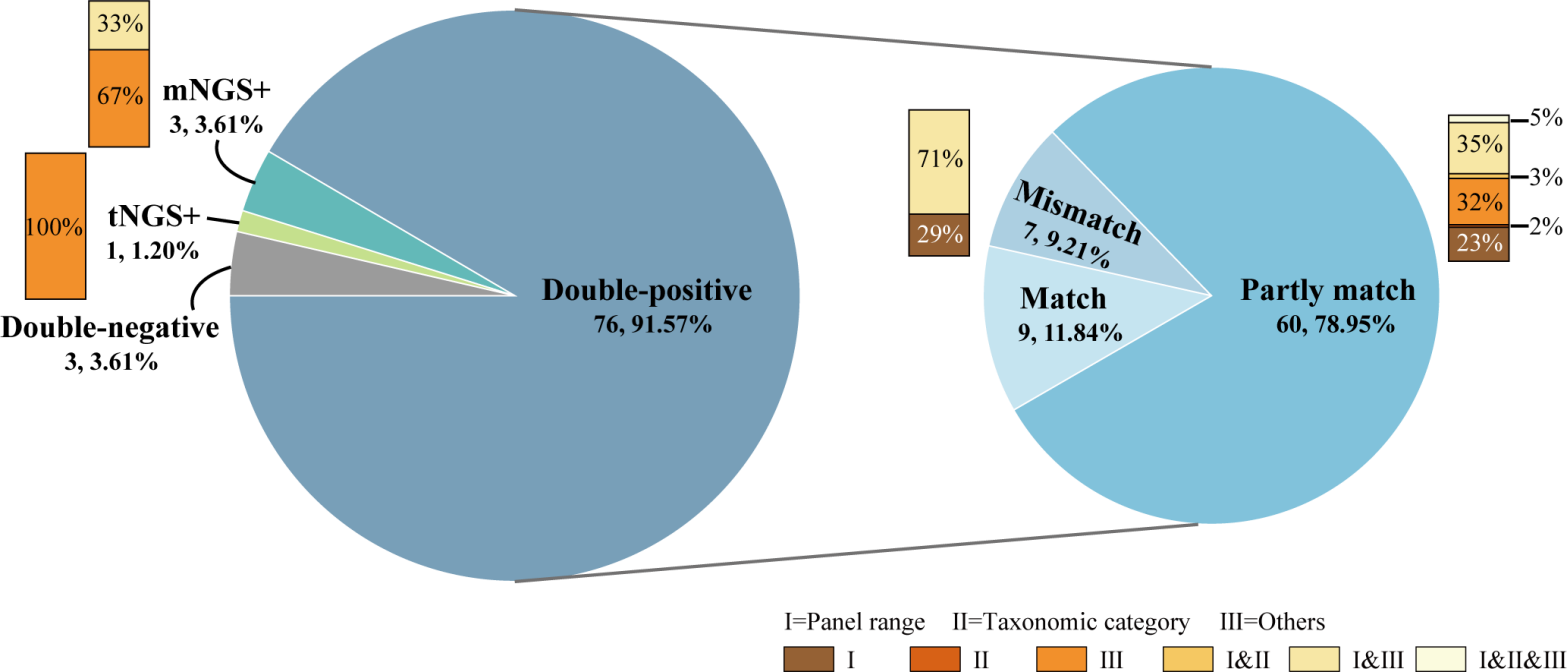
Concordance between mNGS and tNGS assays for pathogen detection.

The concordance between mNGS and tNGS assays for pathogen detection is displayed in **Figure 2**. A total of 76 samples (91.57%, 76/83) were positive for both mNGS and tNGS assays, and 3 (3.61%) were both negative. 9 out of 76 double-positive samples exhibited concordant results between mNGS and tNGS. An additional 60 samples showed partly matched results between mNGS and tNGS, with the same pathogen detected at least once. The remaining 7 samples had discordant results between mNGS and tNGS. Thus, the results of mNGS and tNGS tests on the samples included in this study had a complete consistency rate of 14.56% (12/83), a completely inconsistent rate of 13.25% (11/83), and a partially consistent rate of 72.27% (60/83).

Since the differences between the detection results of mNGS and tNGS workflow were complex, before analyzing the differences in the detection results, we first classified the differences according to the reasons for the inconsistency of the detection results:

I. Different panel ranges: including RNA viruses that couldn’t be detected by the mNGS DNA process, as well as pathogens that couldn’t be detected by the tNGS method without designing primers, but could be detected without bias by mNGS;
II. Different taxonomic categories: in the product design stage, it was determined by many factors such as methodological characteristics, technical accessibility and clinical needs. For example, the mNGS DNA process included and could report *Mycobacterium avium complex* (*MAC*) species such as *M. avium* and *M. intracellulare*, while tNGS only designed universal primers for *MAC*;
III. Other.

Reasons I and II were objective and known. At the same time, there were also uncertain reasons to be explored, that was, category III reasons, such as the degradation of samples due to long-term storage, wet experimental performance, bio-information analysis process, and even interpretation rules. This part was the focus of analysis and future optimization. The classification of the causes of inconsistent detection results helped simplify the difference analysis process and improve the efficiency of difference recognition.

As shown in **Figure 2**, for the 11 cases with no overlapping in the test results, including only mNGS positive, only tNGS positive and mismatch results in the double-positive samples, 2 cases (18.18%) were completely caused by I. different panel ranges, 3 cases (27.27%) were completely caused by III. other factors, and 6 cases (54.55%) had both of the aforementioned two factors. For the partly matched samples among the 60 double-positive samples, 40 cases (66.67%) had different results caused by I. different panel ranges, 6 cases (10.00%) caused by II. different taxonomic categories, and 43 cases (71.67%) caused by III. Other factors. Therefore, the objective and known reasons for the inconsistency between the detection results of mNGS and tNGS workflows in the samples were different panel ranges and different taxonomic categories, which accounted for nearly half of the total, and the other half was caused by differences in methodological performance and other reasons.

**Figure 3:**
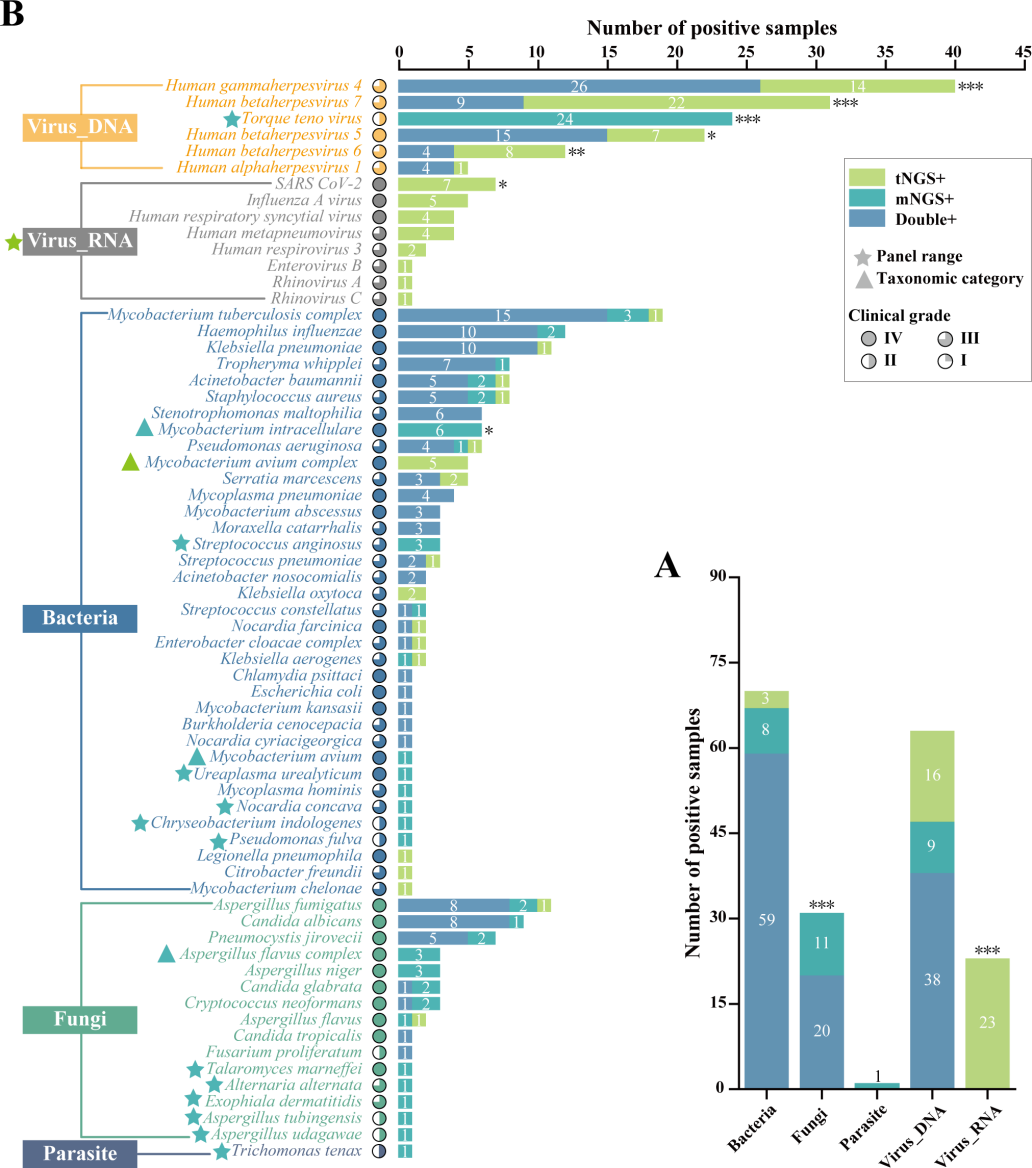
Comprasion of pathogen detection results using mNGS and tNGS assays. **(A)** The bar plot shows the total number of positive samples for each category. **(B)** Pathogens that tested positive either by mNGS or tNGS assays are grouped by biological taxonomy (bacteria, viruses_DNA, viruses_RNA, fungi, and parasites). Blue pentagrams represent mNGS method panel-specific pathogens. Green pentagrams represent tNGS method panel-specific pathogens. Blue triangles represent mNGS method panel-specific taxonomic categories. Green pentagrams represent tNGS method panel-specific taxonomic categories. *P < 0.05; **P < 0.01; ***P < 0.001.

Further, **Figure 3** compared the number of positive samples for mNGS and tNGS detection of each type of pathogen, each pathogen, and specially labeled the pathogens with different panel ranges and different taxonomic categories. The differences in the detection of RNA viruses and parasites were caused by different panel ranges. For bacteria, there was no significant difference in the overall positive rate. Specifically, both workflows detected TOP3 for *MTBC*, *H. influenza*, and *K. pneumonia*, and only the difference in the detection rate of *M. intracellulare* was significant (P<0.05), which was caused by different taxonomic categories. For fungi, the TOP3 fungal detections for both methodologies were *A. fumigatus*, *C. albicans* and *P. jirovecii*. There was no significant difference in the positive rate of pathogens, but there was a higher proportion of specific pathogen detections due to differences in panel ranges and taxonomic categories, which resulted in a significantly higher overall positivity rate for mNGS than for tNGS (P<0.001). For DNA viruses, both workflows detected *Human gammaherpesvirus 4* most. Except for the *Torque teno virus* which was not within the detection range of tNGS, the pathogen spectrum detected by mNGS and tNGS was consistent, which were *Human gammaherpesvirus 4*, *Human betaherpesvirus 5*, *Human betaherpesvirus 7*, *Human betaherpesvirus 6*, *Human alphaherpesvirus 1*. However, the number of positive samples of *Human gammaherpesvirus 4* (P < 0.001), *Human betaherpesvirus 7* (P < 0.001), *Human betaherpesvirus 5* (P < 0.05) and *Human betaherpesvirus 6* (P < 0.01) was statistically different, in which tNGS always had higher detection rates. It was worth noting that this was not due to known objective factors, which may reflect the performance differences between mNGS and tNGS.

In other words, if we excluded the objective and known differences in detection results caused by different panel ranges and different taxonomic categories, only some DNA viruses using tNGS had significantly higher detection rates than mNGS, which implied the detection performance of the two NGS detection methods was similar.

### 3.4 Verification of key pathogens

**Figure 4:**
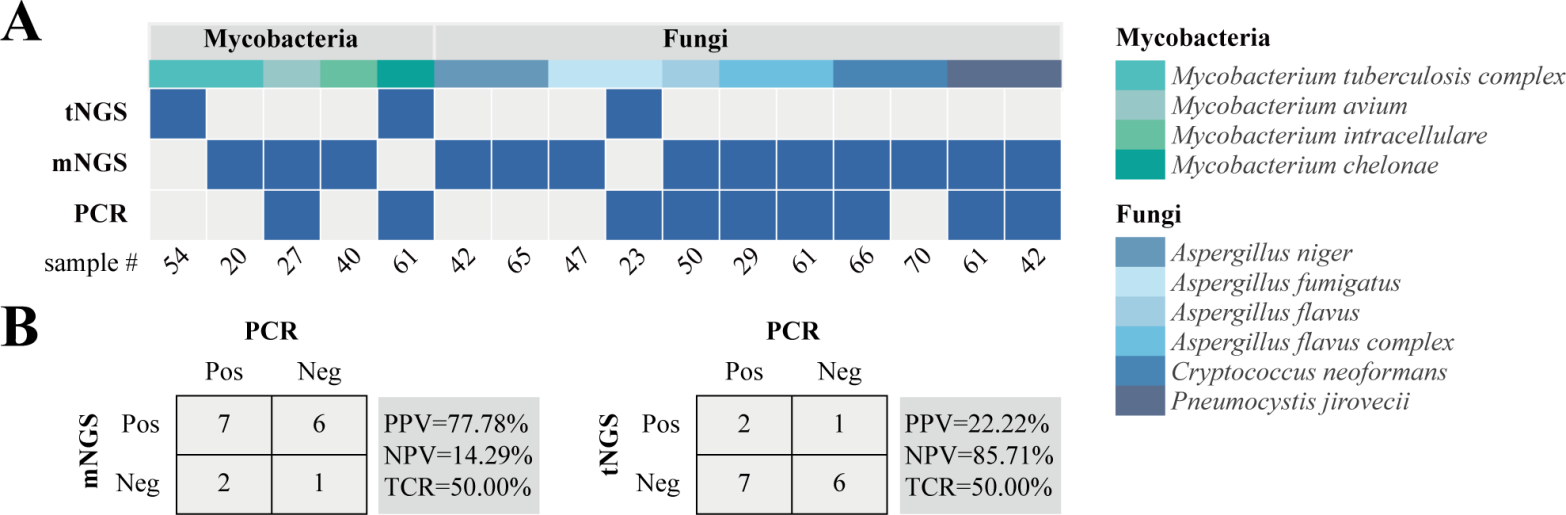
Inconsistent analysis between mNGS and tNGS assays in key pathogens. **(A)** Heat map displaying the result of pathogens detected by tNGS, mNGS, and PCR. **(B)** Contingency tables for the PCR with mNGS and tNGS sets. PPV, positive predictive value; NPV, negative predictive value; TCR, the total coincidence rate.

Based on the results of mNGS and tNGS workflows, PCR orthogonal verification was performed on BALF specimens with differences in clinically critical pathogens. As shown in **Figure 4A**, in the mycobacteria group, 1 sample was detected by mNGS and PCR at the same time, 1 sample was detected by tNGS and PCR simultaneously, and 3 samples were only detected by mNGS or tNGS, which might be due to the instability of the detection caused by the low pathogen load. In the fungal group, 6 samples were simultaneously detected by mNGS and PCR, including 2 cases of *P. jirovecii*, 1 case of *A. flavus*, 2 cases of *A. flavus complex*, and 1 case of *C. neoformans*. One sample was simultaneously detected by tNGS and PCR which was *A. fumigatus*, and the remaining 4 samples were only detected by mNGS including 2 cases of *A. niger*, 1 case of *A. fumigatus*, and 1 case of *C. neoformans*. The positive rate of detection was mNGS > PCR > tNGS. In general, the microbial detection rates of mNGS and PCR were higher than that of tNGS, but the total coincidence rates of mNGS and tNGS with PCR were the same at 50% (**Figure 4B**).

## 4 Discussion

Lower respiratory tract infection (LRTI) is the main cause of death worldwide, particularly among senior citizens in low-income regions, while early and accurate identification of pathogens contributes to decreased morbidity and mortality (Diao et al., 2021; Zheng et al., 2021; Zhang et al., 2022). BAL is a safe, simple and minimally invasive method of sampling the lung microbiome for pathogen diagnosis of lung infections, especially opportunistic lung infections in immunocompromised patients (Zaidi et al., 2020; Xu et al., 2023). However, clinical assays for BALF specimens are inadequate. This study aimed to compare and analyze the detection performance of two emerging NGS-based detection methods, mNGS and tNGS, on clinical BALF samples, and to provide some reference for clinical practice. By analyzing the detection results of mNGS and tNGS workflows for 83 BALF specimens, we found that tNGS may have better detection rates for DNA viruses than mNGS, and the detection performance of the two NGS-based assays for bacteria and fungi was similar, which is comparable to previous studies (Gaston et al., 2022; Li et al., 2022; Huang et al., 2023).

The different taxonomic categories mentioned above, that is, the category II causes of differences in detection results between mNGS and tNGS workflows, are the decision in the workflow design stage. The mNGS method analyzes all microorganisms in the sample without bias in pathogen diagnosis, and there is no targeted enrichment step. Even if the wet experiment stage contains the host-removal step, genomes of the human and background microorganisms also occupy most of the sequencing data. Therefore, this method has the characteristics of a wide detection range but low effective sequencing depth. It may be difficult to distinguish in the face of phylogenetic species with highly similar genomes. Therefore, in the design stage of the mNGS method, we innovatively introduced a “dual categorization level” bio-information analysis process, that is, for the 26 groups of microorganisms in **Supplementary Table 4**, when it is difficult to localize a species by sequence alignment within the group, it is reported at a higher level. For instance, there is a high degree of genomic similarity within *A. flavi*, which possesses both clinically common and rare species, such as *A. flavus*, *A. oryzae*, and *A. alliaceus* (Sabino et al., 2014; Kjærbølling et al., 2020). When the obtained sequences were not sufficient to identify to a species, we reported the *A. flavus complex*, as in sample #29, 43 and 61 (**Supplementary Table 3**). On the other hand, it is well known that the difficulty of the tNGS method based on multiplex PCR lies in the design of primers for the preset targets. In addition to the pursuit of high coverage and high specificity, the more the number of targets, the more serious the interference between primers. Therefore, for species with similar clinical significance and close genetic relationships, the number of targets is reduced and reported uniformly in the design. For example, the biggest cause of nontuberculous mycobacterial lung disease, *M. avium complex* (MAC), includes more than a dozen species such as *M. avium* and *M. intracellulare*, which have similar clinical manifestations and therapeutic measures, and are uniformly reported as MAC, as in sample #38, 44 and 49 (**Supplementary Table 3**) (Griffith, 2018; Liu et al., 2022).

**Figure 5:**
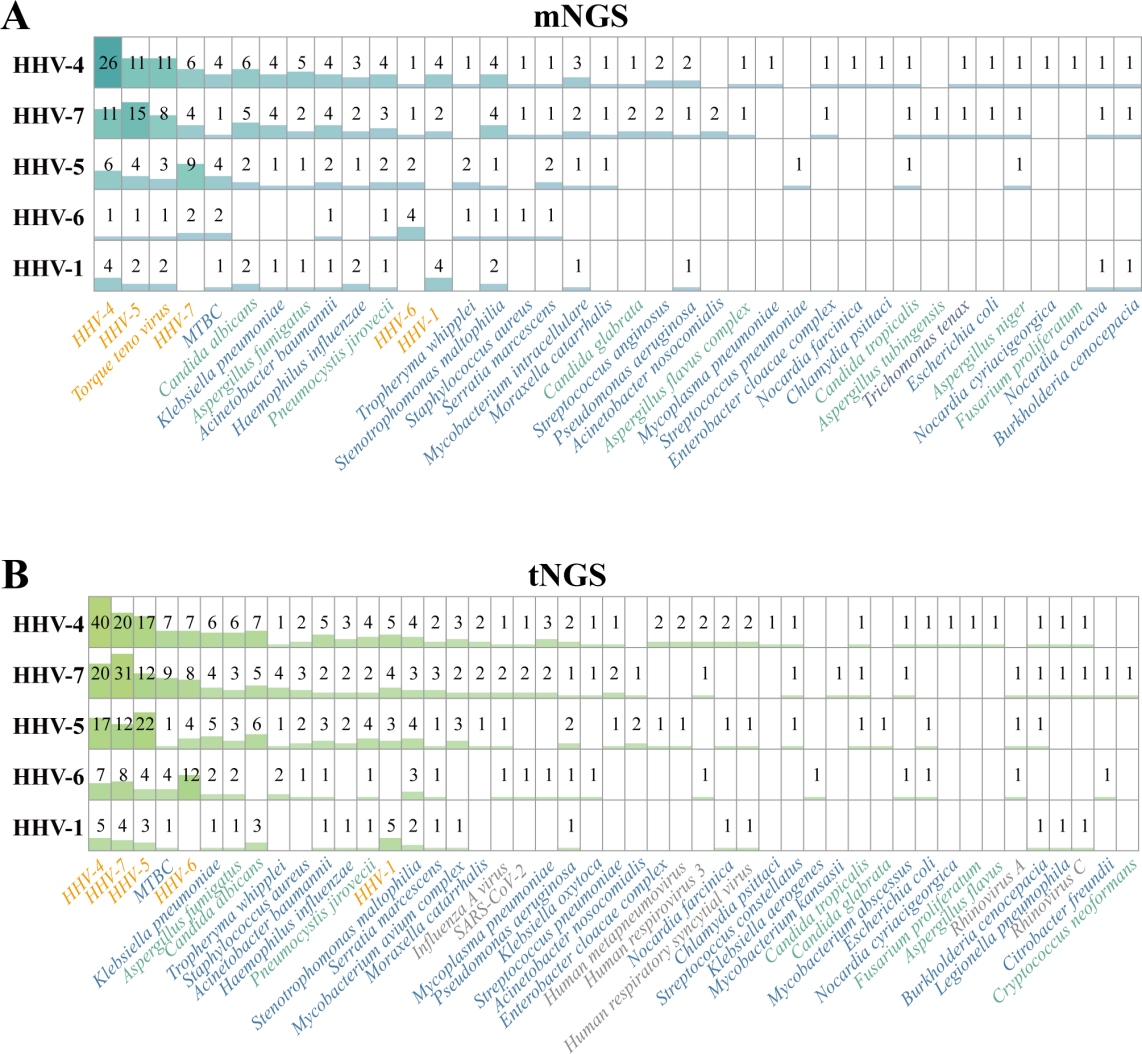
Results of *Human herpesvirus*-positive samples, which detected by **(A)** mNGS and **(B)** tNGS workflows, respectively. *HHV*, *Human herpesviruses*.

In this study, the difference in pathogen detection results of clinical BALF samples between the mNGS and tNGS workflows caused by category III was mainly reflected in the detection of common *Human herpesvirus* (*HHVs*) (**Figure 3B**), that is, tNGS was more sensitive to *HHVs*, which may be due to the targeted enrichment. In order to further verify the credibility of the results, we studied the pathogen detection of *HHVs* detection samples. The detection samples of *HHV-1*, *HHV-4*, *HHV-5*, *HHV-6* and *HHV-7* were traversed, and only one sample was detected as a single herpes virus, that is, only *HHV-4* was detected in Sample #71 by tNGS workflow. In addition, multiple pathogens were detected. The co-infection of *HHVs* detected by tNGS and mNGS is shown in **Figure 5**. Both NGS methodologies show that multiple *HHVs* often co-occur, and *HHVs* are susceptible to co-infection with other pathogens, such as *Torque teno virus*, *C. albicans*, *A. fumigatus*, *P. jirovecii* in fungi, *MTBC*, *A. baumannii*, *K. pneumoniae* in bacteria. This result ties well with previous study, in where applied the mNGS workflow to sputum or BALF from 46 patients with LRTIs, and showed that herpesviruses were frequently detected, the most frequent of which was HHV-4, and was prone to coinfection with *K. pneumonia* and *A. baumannii* (Liu et al., 2022). Another study showed that most samples from lung transplant recipients with unknown etiology of respiratory infection were positive for HHV-7 (Lewandowska et al., 2017). Actually, those HHVs are common opportunistic infections in humans, which might be useful as indicators of the state of host immunity (Walton, 2023).

In this study, the microbial detection rates of mNGS were always equal to or greater than that of tNGS for fungi with the same classification level in the shared detection range of mNGS and tNGS panels (**Figure 3B**). However, there was no statistically significant difference, which may be due to insufficient sample size, low pathogen load and sample degradation. At the same time, it cannot be ruled out whether it is caused by methodological performance differences. In this regard, we also made some attempts, that is, orthogonal verification was performed on 11 clinical key fungi with differences in mNGS and tNGS results (**Figure 4A**). The results showed that the consistency rate of mNGS and PCR results (54.55%) was only slightly higher than that of tNGS and PCR results (45.45%). Subsequent attention should be paid to the detection performance of mNGS and tNGS for fungi.

The comparison of the results of mNGS and tNGS workflows can expose the primer design flaws of the primers for the tNGS process. For this study, in Sample #49, mNGS detected *Nocardia concava*, and tNGS detected *N. farcinica*, which was *N. concava* by sequence alignment, that is, the primers designed for *N. farcinica* by tNGS workflow mistakenly amplified the *N. concava* sequence. Coincidentally, in Sample #23, the primers designed for *A. fumigatus* in the tNGS workflow mistakenly amplified the *A. tubingensis* sequence. These two examples are caused by the lack of specificity of primers between the phylogenetic species, and the subsequent optimization of the corresponding primers needs to be completed, or whether the minimum common taxonomic unit can be reported through its clinical significance evaluation.

In this study, both NGS workflows show their advantages of high sensitivity and wide detection range in pathogen detection, which helps assist clinical diagnosis. Taking mycobacteria as an example, Sample #6 was clinically diagnosed as suspected pulmonary tuberculosis, while both mNGS and tNGS detected *M. kansasii* but not *MTBC*, indicating that the NGS methods can effectively distinguish *MTBC* from non-tuberculous *Mycobacteria* (*NTM*). This has important clinical significance because treatments of TB and NTM diseases are different (Gopalaswamy et al., 2020). Similarly, for Sample #55, both NGS workflows detected MAC and *M. abscessus*. Mixed infections between *NTMs* are easily missed by clinical or other detection methods, but in fact, there are differences in clinical treatment options for the two (Daley et al., 2020).

For the two NGS workflows, except for the difference in detection results, the price of tNGS is lower than mNGS. Because tNGS needs to enrich the target, its wet experiment process is more lengthy, and turnaround time (TAT) is slightly longer than mNGS single process. However, because tNGS can simultaneously detect DNA and RNA, it has an advantage over mNGS in terms of operational complexity. It should be noted that cost and TAT were defined as the cost and time incurred from the beginning of DNA extraction to the time when the result is obtained.

In addition, tNGS based on multiplex PCR technology can only detect the target pathogens with preset primers, which are currently common in tens to hundreds of pathogens (Chao et al., 2020; Huang et al., 2021; Li et al., 2023). mNGS is capable of unbiased detection, covering a wide range of pathogens, and even detecting new pathogens (Chiu and Miller, 2019). Based on the above factors, the two NGS methods can complement each other. mNGS is more suitable for critical infections, especially for unexplained infections. tNGS is more suitable for moderate and critical infection, and non-specific pathogen infection.

This study had some limitations as follows: (i) Fresh BALF samples were used for mNGS detection, and frozen samples in the biological sample bank were used for tNGS detection. At the same time, the number of samples is small; (ii) The detection results lack clinical prognosis evaluation; (iii) The types of available PCR kits were limited. In addition, some samples are not enough to be verified by PCR.

## 5 Conclusion

This study systematically evaluated the efficacy differences between mNGS and multiplex PCR-based tNGS in BALF specimens. In general, mNGS and tNGS have similar effects on the detection of bacteria and fungi, while tNGS may be superior to mNGS in the detection of DNA viruses. There was no significant difference between the two NGS assays compared to PCR. Our results provide a reference for the clinical application of mNGS and tNGS.

## Data Availability

The datasets presented in this study can be found in online repositories. The names of the repository/repositories and accession number(s) can be found below: https://db.cngb.org/cnsa/, CNP0005812.
